# Association of Serotonin_2c_ Receptor Polymorphisms With Antipsychotic Drug Response in Schizophrenia

**DOI:** 10.3389/fpsyt.2019.00058

**Published:** 2019-02-15

**Authors:** Jiang Li, Hitoshi Hashimoto, Herbert Y. Meltzer

**Affiliations:** ^1^Department of Psychiatry and Behavioral Sciences, Northwestern University Feinberg School of Medicine, Evanston, IL, United States; ^2^Laboratory of Molecular Neuropharmacology, Graduate School of Pharmaceutical Sciences, Osaka University, Suita, Japan; ^3^Molecular Research Center for Children's Mental Development, United Graduate School of Child Development, Osaka University, Kanazawa University, Hamamatsu University School of Medicine, Chiba University and University of Fukui, Osaka University, Suita, Japan; ^4^iPS Cell-based Research Project on Brain Neuropharmacology and Toxicology, Graduate School of Pharmaceutical Sciences, Osaka University, Suita, Japan; ^5^Division of Bioscience, Institute for Datability Science, Osaka University, Suita, Japan; ^6^Transdimensional Life Imaging Division, Institute for Open and Transdisciplinary Research Initiatives, Osaka University, Suita, Japan

**Keywords:** serotonin_2C_, schizophrenia, genetic, treatment response, antipsychotic agents, clozapine, meta-analysis, polymorphism

## Abstract

There is conflicting evidence for the association between genetic polymorphisms in the serotonin (5-HT)_2C_ receptor (HTR2C) and response to antipsychotic drugs (APD) in schizophrenic patients. We tested the association between the HTR2C polymorphisms, Cys23Ser, −759C/T, and −697G/C, and response to APDs (mainly clozapine) in a 6 month prospective study in 171 patients with schizophrenia. Ser23 was significantly associated with treatment response (positive symptoms, *X*^2^ = 7.540, *p* = 0.01; negative symptoms, *X*^2^ = 4.796, *p* = 0.03) in male patients only. A −759C-Ser23 haplotype was similar associated with positive (*X*^2^ = 6.648, *p* = 0.01) and negative (*X*^2^ = 6.702, *p* = 0.01) symptom improvement. Logistic regression, after controlling for covariates, also showed significant haplotypic associations. A meta-analysis of six studies for Ser23 and treatment response showed an overall odds ratio of 2.00 (95%CI, 1.38–2.91, *p* = 0.0003) or 1.94 (95%CI, 1.27–2.99, *p* = 0.0024) under fixed or random effect models. These results provide additional evidence that HTR2C polymorphisms are associated with treatment response to APD with HTR2C antagonism or inverse agonism, in male schizophrenic patients.

## Introduction

The serotonin (5-HT)_2C_ receptor (HTR2C), located at Xq24, belongs to the seven-transmembrane-spanning G protein–coupled receptor superfamily. It is widely distributed in brain regions which are relevant to schizophrenia. HTR2C receptors exert a tonic inhibitory effect on dorsal and ventral striatal, limbic, hippocampal, and cortical dopamine (DA) release (1, 2), modulate serotonergic activity in the dorsal raphe (3), and regulate 5-HT and glutamate efflux in rat cortex (4).

HTR2C is involved in the neurobiology of schizophrenia and the efficacy and side effects of some APDs. It is one of the key regulators of dopaminergic activity in the limbic system. Stimulation of DA D2 receptors in the ventral and dorsal striatum can lead to delusions and hallucinations (5). Activation of HTR2C receptors might lead to decreases in DA release in key brain regions for schizophrenia (6). HTR2C are expressed on principal neurons and GABAergic interneurons in the prefrontal cortex (7) and, thus, may be relevant to the hypoglutamatergic basis for various components of the schizophrenia syndrome (8). The ability of 5-HT- stimulated and constitutively active HTR2C receptors to inhibit DA release in limbic brain areas has been postulated to cause psychosis and to modulate the efficacy of APDs that act by blocking DA D2 receptors (9–12). Blockade of the constitutive activity of HTR2C receptors enhances cortical and limbic DA release by some APDs (12). The ability of HTR2C agonists to reduce DA release from terminals of VTA neurons in mesolimbic areas is consistent with the antipsychotic effect of the HTR2C agonist, vabicaserin (13, 14). Vabicaserin is effective in reversal of phencyclidine and amphetamine-induced hyperactivity (15).

**Figure 1:**
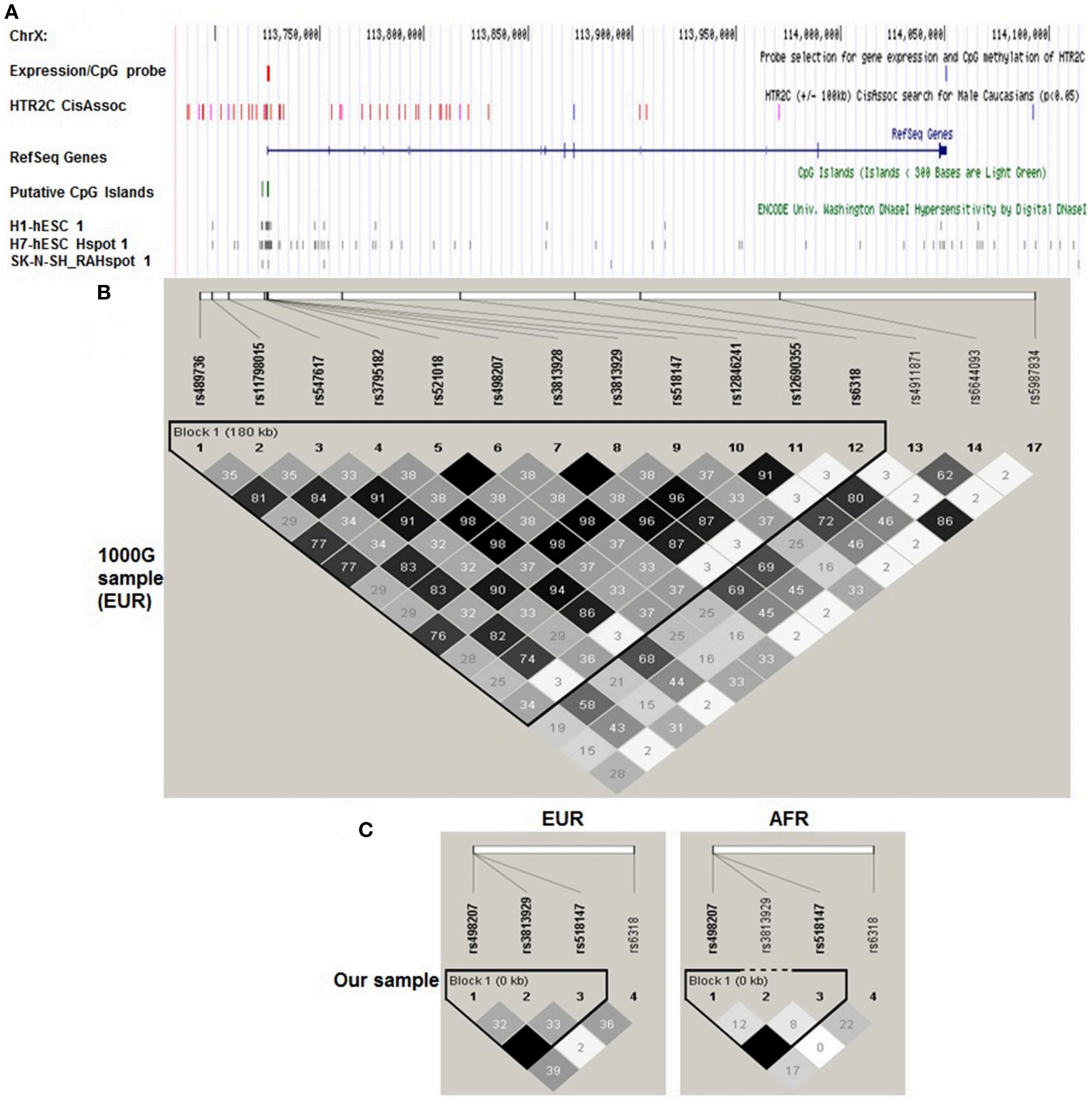
Haploview plot of cis-eQTLs identified by Braincloud for HTR2C in EUR. A. Multiple custom annotation tracks were created at UCSC Genome Browser to show the *cis* eQTLs for HTR2C. “expression/CpG Probe” track represents the probe location for gene expression (blue) and CpG island (red); **(A)** Single expression probe selected for HTR2C is located at the 3′ end of mRNA which represents the major form of HTR2C cloned from multiple brain regions according to NCBI Aceview. “HTR2C CisAssoc” track represents identified cis-eQTLs (*p* < 0.05) by Braincloud data (purple) and all other SNPs in LD (*r*^2^ > 0.8) with cis-eQTLs (red). We successfully identified the tag SNPs for the candidate SNPs (*r*^2^ > 0.85) by SNAP, based on earlier Hapmap data (16) in EUR. rs12846241 (*r*^2^ = 0.96), rs498207 (*r*^2^ = 0.98), and rs5987834 (*r*^2^ = 0.86) are the tag SNPs, genotyped in Braincloud, for −759C/T, −697G/C, and Cys23Ser, respectively, in EUR. Most of *cis*-eQTLs are aggregated at the promoter or 5′ UTR regions as expected. −759C/T, tagged by rs12846241, has been confirmed as *cis*-eQTL for HTR2C. The T genotype has a lower mRNA level than C genotype (*p* = 0.006). −697G/C, tagged by rs498207, also suggests a potential impact on gene expression (*p* = 0.043), but not as strong as −759C/T. Cys23Ser and its tag SNP rs5987834 were negative controls (blue); “CpG Islands” track represents putative CpG Islands for HTR2C. We also include putative *cis* regulatory elements identified by DNase I hypersensitivity analysis on several cell lines with potential neuronal lineage. **(B)** A haploview plot of those SNPs in EUR from 1,000 Genome data. Grayscale from black to white is correlated with r^2^ from high to low in each box. **(C)** Haploview plot of genotyped HTR2C SNPs in our sample separated by ethnicity. The LD and haplotype pattern of genotyped SNPs from our EUR samples matched those from 1,000 Genome EUR sample.

Evidence from genetic association studies also implicates HTR2C in a variety of neuropsychiatric diseases. The HTR2C has a well-characterized promoter region harboring multiple polymorphisms (Figure 1A), suggesting their potential impact on CpG methylation and putative transcription factor binding, resulting in alteration of HTR2C expression. −759C/T and −697G/C are the most widely investigated promoter polymorphisms. −759C/T polymorphism is associated with antipsychotic induced weight gain (17). −759C/T or −697G/C, has also been linked to therapeutic response to APDs (18–20). However, these results are contradictory to each other with regard to gender and risk allele.

Cys23Ser, is a non-synonymous SNP which results in an amino acid substitution of cysteine to serine at position 23. This substitution can disrupt a disulfide bridge and potentially alter the structure or stability of the HTR2C protein (21). Although this functional polymorphism has been found to be associated with numerous neuropsychiatric diseases, including anorexia nervosa (22), unipolar, and bipolar depression (16, 23), psychotic symptoms in late-onset Alzheimer's disease (24), vulnerability to cocaine cue reactivity (25), migraine with aura (26), and stress-related cortisol levels (27), it's association with schizophrenia is less clear (28). An association between the Cys23Ser and visual hallucinations and depression in schizophrenia patients has been reported (24), but has not been replicated by others (29, 30). Cys23Ser is associated with chronic hospitalization in schizophrenia patients (31) and APD-induce extrapyramidal side effects (32) suggests it is more related to the impact of treatment on the disease process, possibly through effects on dopaminergic activity. Most importantly for this study, Cys23Ser has also been linked in some (33, 34) but not all studies (35–37) to the extent of response to clozapine. These studies will be the subject of a meta-analysis included in the Results section. Clozapine response in treatment-resistant schizophrenia patients does not occur within the conventional 6 week clinical trial period in many patients (38, 39). Due to the inconsistent relationship between HTR2C polymorphisms and the psychopathology of schizophrenia, the response to clozapine, and functional activity assays, we examine all three widely-investigated HTR2C SNPs as possible predictors of response of positive and negative symptoms to APD treatment in schizophrenia. Male and female subjects were analyzed separately in order to determine a possible association with gender. Finally, a meta-analysis was performed to determine the overall association between HTR2C polymorphisms and response to clozapine and other APDs.

## Materials and Methods

### Subjects

The 171 (male/female, 115/56) patients with schizophrenia or schizoaffective disorder who participated in this study were part of an NIMH-sponsored extramural clinical research center at Case Western Reserve University School of Medicine and Vanderbilt University School of Medicine. Details about recruitment and assessment of subjects have been reported previously (40). Categorical treatment response was evaluated at 6 week and 6 months, using the criteria based upon Kane et al. (41). Subjects with a reduction of >20% in the Brief Psychiatric Rating Scale (BPRS) total score or subcategories, BPRS Positive and Negative, was defined as a responder. Patients were treated with standard doses of the following atypical antipsychotic drugs: clozapine, 550 mg(400–900 mg), 78%; melperone, 250 mg(100–400 mg), 7.0%; risperidone, 6 mg(4–8 mg), 3.8%; or olanzapine, 20 mg(15–40 mg), 2.1%, or typical antipsychotic drugs, mainly haloperidol (10 mg, 9.0%). Antidepressants (14%) and mood stabilizers (5%) were used sparingly.

### Genotyping

Taqman® assay for three SNPs, −759C/T(rs3813929), −697G/C(rs518147), and Cys23Ser(rs6318) was performed at Northwestern University Genomic Core. Call rates are 95.32, 98.83, and 97.66%, respectively. The linkage disequilibrium (LD) and haplotype pattern of genotyped SNPs from our EUR samples (Figure 1C) matched those from 1,000 Genome EUR sample (Figure 1B).

### Statistical Analysis (see Supplementary Information for Details)

We analyzed the males and females separately. The relationship between genotypes and demographic variables was analyzed using chi-square (χ^2^) or ANOVA. Genotype or haplotype associated differential response to APD was initially evaluated by χ^2^ test and then ANCOVA (SPSS), adjusted for race, drugs, age of onset, and the corresponding baseline psychopathology or status of early response. Statistical significance was defined as *p* < 0.05. As all results were considered exploratory, there was no adjustment for multiple testing. Mapping cis eQTL or methylation QTL was performed using Braincloud data (42, 43). In order to review and elucidate the general relationship between HTR2C polymorphisms and drug response to APDs, a meta-analysis of six studies, including ours, with accessible genotyping data for Cys23Ser and binary outcome for symptom improvement, were conducted by R “meta” package. Heterogeneity among the studies was assessed by means of the *I*^2^ inconsistency test and Cochran's *Q* statistics under a null hypothesis test in which *p* < 0.05.

## Results

### Demographic Data Grouped by Genotypes of Three HTR2C SNPs

Cys23Ser, −759C/T, and −697G/C were genotyped for schizophrenic patients with European (EUR, *n* = 118) and African (AFR, *n* = 53) ancestry Table 1. In the male group, the age at onset for the Ser23 carriers was significantly older than that for non-carriers (*p* = 0.009; Table 1A). This difference was not observed in the females (*p* = 0.899; Table 1B). There was no significant difference in the proportions of patients who were treatment resistant or unmedicated at baseline between the genotypes for each SNP. Duration of illness and number of previous hospitalization also did not differ. Although Ser23 carriers had a higher total BRPS score in the male patients (*p* = 0.04), there was no significant difference with regard to the subcategories of psychopathology including positive, negative, and anxiety/depression subscales. Race, drug, age of onset, and baseline psychopathology were included as covariates in the following ANCOVA.

Table 1Demographic information grouped by genotypes of three HTR2C SNPs and separated by gender (1A for Male; 1B for Female).

**Table:** 

**SNP ID**	**-759C/T(rs3813929)**			**-697G/C(rs518147)**			**Cys23Ser(rs6318)**		
**Genotype**	**T/T**	**C/C**	***P*-value**	**C/C**	**G/G**	***P*-value**	**Ser23 carrier**	**Ser23 non-carrier**	***P*-value**
**A**									
Counts	17	100		40	77		24	90	
Frequency	0.14	0.86		0.34	0.66		0.22	0.78	
Total BPRS	27.88 ± 17.51	30.57 ± 12.08	0.43	32.3 ± 14.9	29.08 ± 11.78	0.2	34.92 ± 12.30	29.03 ± 12.89	0.04
Positive 4 items	9.41 ± 7.20	11.22 ± 5.36	0.23	11.4 ± 5.74	10.73 ± 5.65	0.54	12.69 ± 4.14	10.59 ± 5.81	0.09
Positive 3 items	7.47 ± 5.91	9.04 ± 4.72	0.23	9.282 ± 4.91	8.55 ± 4.94	0.45	10.25 ± 3.53	8.57 ± 5.04	0.13
Negative 3 items	3.94 ± 2.33	4.43 ± 3.01	0.53	4.15 ± 2.94	4.47 ± 2.91	0.58	4.65 ± 3.03	4.30 ± 2.87	0.59
Anxiety/Depression	4.69 ± 2.87	4.99 ± 3.67	0.75	5.39 ± 3.49	4.72 ± 3.59	0.35	5.24 ± 3.70	5.00 ± 3.48	0.72
Age of onset (year)	20 ± 6.24	20.26 ± 5.12	0.86	20.75 ± 6.36	19.93 ± 4.60	0.43	22.78 ± 7.83	19.57 ± 4.27	0.01
Duration of illness (year)	11.71 ± 11.34	12.21 ± 7.45	0.81	11.63 ± 9.50	12.41 ± 7.26	0.62	10.61 ± 8.63	12.42 ± 8.01	0.34
No of previous hospitalization	4.38 ± 4.24	6.66 ± 7.15	0.22	4.54 ± 4.09	7.28 ± 7.78	0.05	4.76 ± 4.13	6.56 ± 6.96	0.26
Treatment resistant (%)	52.9	70.0	0.17	60	71.4	0.21	62.5	68.9	0.55
Baseline Unmedicated (%)	81.3	76.9		78.4	77.1	0.88	72.7	78.0	0.6
Clozapine treated (%)	82.4	74.0	78.7	85.0	72.7	0.34	83.3	74.4	0.28
Ethnicity^a^			0.004			0.19			0.04
EUR	12	67		24	55		11	66	
AFR	5	33		16	22		13	24

**Table:** 

**SNP ID**	**-759C/T(rs3813929)**			**-697G/C(rs518147)**				**Cys23Ser(rs6318)**		
**Genotype**	**T/T**	**C/C**	***P*****-value**	**C/C**	**C/G**	**G/G**	***P*****-value**	**Ser23 carrier**	**Ser23 non-carrier**	***P*****-value**
**B**										
Counts	3	41		7	24	21		20	33	
Frequency	0.07	0.93		0.13	0.46	0.4		0.38	0.62	
Total BPRS	28.33 ± 9.29	34.27 ± 11.48	0.39	30.86 ± 11.85	35.71 ± 11.75	33.81 ± 10.61	0.8	34.9 ± 14.41	33.82 ± 8.40	0.73
Positive 4 items	8.33 ± 6.11	12.66 ± 4.52	0.12	11.14 ± 6.67	13.58 ± 4.07	12 ± 4.89	0.91	12.9 ± 5.18	12.15 ± 4.76	0.58
Positive 3 items	7 ± 5.29	11.03 ± 3.93	0.1	10 ± 6.83	11.78 ± 3.57	10.43 ± 4.06	0.83	11.2 ± 4.95	10.5 ± 4.2	0.58
Negative 3 items	3.33 ± 3.22	4.39 ± 3.31	0.6	4 ± 3.74	4.5 ± 2.70	4.76 ± 3.90	0.61	4.43 ± 3.01	4.62 ± 3.46	0.84
Anxiety/Depression	7.33 ± 4.73	6.42 ± 4.12	0.71	6.14 ± 3.53	5.75 ± 3.92	6.91 ± 4.44	0.48	6.05 ± 3.94	6.27 ± 4.03	0.85
Age of onset (year)	20.33 ± 4.04	21.55 ± 5.85	0.73	21 ± 4.08	21.30 ± 5.47	21.29 ± 5.68	0.99	21.79 ± 4.85	21.58 ± 6.27	0.9
Duration of illness (year)	14 ± 15.59	11.63 ± 7.27	0.62	15.29 ± 11.41	14.74 ± 7.81	10 ± 6.51	0.1	13.05 ± 6.96	12.49 ± 8.73	0.81
No of previous hospitalization	3 ± 2.83	8.11 ± 8.74	0.42	4.5 ± 3.14	7 ± 5.51	9.4 ± 10.21	0.35	7.94 ± 9.50	7.03 ± 6.79	0.71
Treatment resistant (%)	66.7	70.7	0.88	71.4	79.2	61.9	0.44	80	69.7	0.41
Baseline Unmedicated (%)	100.0	63.6	0.29	66.7	78.9	61.1	0.49	60.0	72.4	0.4
Clozapine treated (%)	66.7	85.4	0.1	71.4	87.5	90.5	0.003	90.9	75.0	0.14
Ethnicity^a^			0.2				0.62			0.08
EUR	3	26		6	16	15		12	27	
AFR	0	15		1	8	6		8	6	

The relationship between genotypes and demographic variables was analyzed using chi-square (χ^2^) or ANOVA. p-values reported are two tailed whenever applicable. Statistical significance was defined as p < 0.05.

a *Self-described ethnicity*.

### Genotype/Haplotype Associated Differential Response to APDs

We successfully identified the tag SNPs for the candidate SNPs (*r*^2^ > 0.85) by SNAP and these tag SNPs are in LD with the three candidates by a haploview analysis of the genotype data from 1,000 Genome Figure 1, Supplementary Table 1. −759C/T, tagged by rs12846241, have been confirmed as *cis*-eQTL and methylation-QTL for HTR2C (Supplementary Table 2). Cys23Ser, tagged by rs5987834, has no impact on gene expression and % methylation. Based on the previous studies (genetic and functional) and our *cis* eQTL findings, −759C/T, −697G/C, Cys23Ser, and the combinations of two or all three, were targets for the subsequent genotype-phenotype association study. Since *in vitro* functional assays indicated that −759C/T and Cys23Ser have a significant impact on HTR2C activity through distinctive mechanisms, we further explored if −759C-Ser, “a super combination,” we propose it produces a greater expression of the constitutively more active form of HTR2C, may demonstrate an even stronger association with dichotomous symptom improvement in an additive mode, after treatment with the APDs, which are HTR2C inverse agonists or antagonists studied here.

A significant association between Cys23Ser and dichotomous treatment response was observed only in the male group for both positive symptoms, *X*^2^ = 7.540, *p* = 0.006; and negative symptoms, *X*^2^ = 4.796, *p* = 0.029, at 6 month (Table 2A). Haplotype analysis showed that −759C-Ser23 maintained the same level of significant association with positive symptom improvement (*X*^2^ = 6.648, *p* = 0.010) and negative symptom improvement (*X*^2^ = 6.702, *p* = 0.010) at 6 month (Table 2A). All of the above significant findings were only observed in the male patients, except for a borderline significance for Cys23Ser associated with negative symptom improvement in female (*X*^2^ = 3.9, *p* = 0.048) at 6 month (Supplementary Table 3).

Table 2Cys23Ser(rs6318) is associated with differential treatment response in male patients.

**Table:** 

**SNP ID**	**Haplotype**	**Haplotype frequency**	**BPRS positive 4 items**		**BPRS positive 3 items**		**BPRS negative 3 items**	
			**Frequency in responder/non-responder**	***X*^**2**^*/P***	**Frequency in responder/non-responder**	***X*^**2**^*/P***	**Frequency in responder/non-responder**	***X*^**2**^*/P***
**A**								
**MALE ONLY**								
rs3813929 (-759)	C	0.861	0.81/0.88	0.86/0.353	0.85/0.85	0/1	0.84/0.82	0.06/0.807
rs518147 (-697)	C	0.336	0.45/0.26	3.84/0.050	0.45/0.26	3.78/0.052	0.41/0.29	1.30/0.255
rs6318	Ser carrier	0.22	0.29/0.16	2.41/0.121	0.34/0.11	7.54/0.006	0.31/0.13	4.80/0.029
rs3813929-rs518147	C-C	0.197	0.26/0.14	2.30/0.129	0.30/0.11	5.34/0.021	0.24/0.11	2.92/0.088
rs518147-rs6318	C-Ser	0.176	0.24/0.13	2.32/0.128	0.28/0.09	6.07/0.014	0.24/0.09	3.83/0.050
rs3813929-rs6318	C-Ser	0.202	0.27/0.16	1.67/0.196	0.33/0.11	6.65/0.010	0.31/0.09	6.70/0.010
rs3813929-rs518147-rs6318	C-C-Ser	0.171	0.24/0.12	2.26/0.133	0.28/0.09	5.82/0.016	0.22/0.09	3.06/0.080

**Table:** 

**SNP ID**	**-759C/T(rs3813929)**			**-697G/C(rs518147)**				**Cys23Ser(rs6318)**		
**Genotype**	**T/T**	**C/C**	**F/P**^**&**^	**C/C**	**C/G**	**G/G**	**F/P**	**Cys/Ser+Ser/Ser**	**Cys/Cys**	**F/P**
**B**										
**MALE ONLY**										
Counts	17	95		39	NA	73		24	89	
Frequency	0.1404	0.8596		0.3421	NA	0.6579		0.2174	0.7826	
Total_6Mon	−8.47 ± 13.25	−7.06 ± 12.08	0.748/0.389^#^	−10.36 ± 11.62	NA	−5.64 ± 12.30	1.30/0.256	−12.65 ± 9.09	−6.18 ± 12.60	2.82/0.096
Total_6Wk	−7.00 ± 8.23	−5.63 ± 11.92	3.93/0.05^#^	−8.25 ± 11.13		−5.39 ± 12.57	0.67/0.413	−9.58 ± 11.63	−6.20 ± 12.12	0.08/0.784
Positive 4 items_6Mon	−2.06 ± 4.66	−2.65 ± 5.75	0.01/0.914^#^	−3.56 ± 4.77	NA	−2.01 ± 5.92	0.99/0.322	−5.22 ± 5.04	−2.09 ± 5.66	3.86/0.052
Positive 4 items_6Wk	−2.76 ± 4.66	−2.03 ± 5.27	1.99/0.161	−2.83 ± 4.60		−1.77 ± 5.45	0.92/0.339	−3.19 ± 4.52	−2.03 ± 5.39	0.08/0.772
Positive 3 items_6Mon	−1.35 ± 4.36	−2.25 ± 4.77	0.04/0.835	−2.97 ± 4.33	NA	−1.61 ± 4.86	0.82/0.367	−4.74 ± 4.26	−1.59 ± 4.77	5.19/0.025
Positive 3 items_6Wk	−2.24 ± 4.31	−1.61 ± 4.23	1.81/0.181	−2.33 ± 4.10		−1.36 ± 4.28	1.08/0.302	−2.62 ± 3.97	−1.59 ± 4.37	0.14/0.705
Negative 3 items_6Mon	−0.41 ± 3.87	−0.85 ± 2.75	0.15/0.700	−1.25 ± 3.62	NA	−0.52 ± 2.51	2.64/0.107	−2.04 ± 2.79	−0.52 ± 2.99	5.73/0.019
Negative 3 items_6WK	0.00 ± 2.78	−0.15 ± 2.84	0.14/0.708	−0.03 ± 2.36		−0.18 ± 3.05	0.05/0.819	−0.54 ± 2.21	−0.14 ± 3.06	0.67/0.416
**FEMALE ONLY**										
Counts	3	40		7	23	21		20	34	
Frequency	0.06818	0.9318		0.1346	0.4615	0.4038		0.3818	0.6182	
Total_6Mon	−7.33 ± 8.33	−9.81 ± 10.82	0.29/0.595	−9.71 ± 14.10	−9.88 ± 10.53	−11.17 ± 11.15	0.22/0.802	−10.31 ± 13.28	−11.45 ± 9.93	0.06/0.811
Total_6Wk	−3.33 ± 3.22	6.68 ± 12.19	0.37/0.0.549^#^	−9.43 ± 7.85	−8.32 ± 14.05	−7.76 ± 11.84	0.24/0.788	−9.73 ± 12.97	−8.00 ± 12.20	0.22/0.641
Positive 4 items_6Mon	0.33 ± 6.11	−3.75 ± 4.10	1.90/0.178	−2.43 ± 5.41	−3.71 ± 3.62	−4.33 ± 4.83	0.74/0.483	−3.31 ± 3.61	−4.10 ± 4.64	0.44/0.512^#^
Positive 4 items_6Wk	0.33 ± 5.77	−2.55 ± 4.44	0.92/0.344	−3.71 ± 5.74	−2.56 ± 4.74	−2.76 ± 4.00	0.70/0.502	−3.41 ± 4.35	−2.56 ± 4.47	0.45/0.506
Positive 3 items_6Mon	0.33 ± 4.51	−3.42 ± 3.64	2.00/0.168	−2.14 ± 5.34	−3.00 ± 3.37	−4.06 ± 4.15	1.19/0.315	−2.53 ± 3.74	−3.59 ± 4.06	1.05/0.311^#^
Positive 3 items_6Wk	1.00 ± 5.29	−2.22 ± 4.04	1.41/0.242	−3.00 ± 5.69	−1.78 ± 4.59	−2.71 ± 3.24	1.03/0.366	−2.55 ± 4.19	−2.24 ± 4.01	0.11/0.739
Negative 3 items_6Mon	−1.67 ± 3.06	−0.59 ± 2.45	0.73/0.400	−1.57 ± 5.53	−0.53 ± 2.40	−0.61 ± 3.15	0.81/0.454	−1.75 ± 4.09	−0.38 ± 2.90	2.46/0.124
Negative 3 items_6WK	1.33 ± 2.31	−0.93 ± 2.62	2.03/0.162	0.14 ± 2.91	−1.00 ± 2.68	−1.14 ± 2.83	0.49/0.617	−1.45 ± 2.86	−0.62 ± 2.78	2.26/0.139

A. Haplotype association analysis of HTR2C SNPs with treatment response at 6mon by Chi-square. Positive 4-item includes suspiciousness, hallucinatory behavior, unusual thought content, and conceptual disorganization. Positive 3-item = Positive 4-item without “concept disorganization.” Negative 3 items includes emotional withdrawal, motor retardation, and blunted affect; Male and female subjects were analyzed separately. B. Ser23 carriers have a better symptom improvement in male subjects. All data was presented as Mean ± SD for Δ change (absolute change), which is calculated by (6 week or 6 month-Baseline);

& represents F statistic and p-value calculated from ANCOVA on Δ change at 6 week or 6 month after controlling for race, drugs, age of onset, and the corresponding baseline psychopathology.

# *represents the result from Levene's test of equality of error variances with p < 0.05, which against the null hypothesis that the error variance of the dependent variable is equal across groups, suggesting ANCOVA assuming homogeneity of variance is rejected*.

ANCOVA test on absolute change (Table 2B) or % change in BPRS (data not shown) in symptom improvement after controlling for race, drugs, age of onset, and the corresponding baseline psychopathology indicated that male Ser23 carriers had a significant improvement in positive and negative symptoms (*p* = 0.025 and 0.019, respectively) after 6 months treatment (Table 2B). Neither −759C/T nor −697G/C alone were significantly associated with symptom improvement.

A similar significant association was observed between Cys23Ser and positive/negative symptom improvement in male subjects treated with clozapine only (Supplementary Table 4). Female Ser23 carriers also showed an association with negative symptom improvement at 6 week.

### Meta-analysis and Power Test

**Figure 2:**
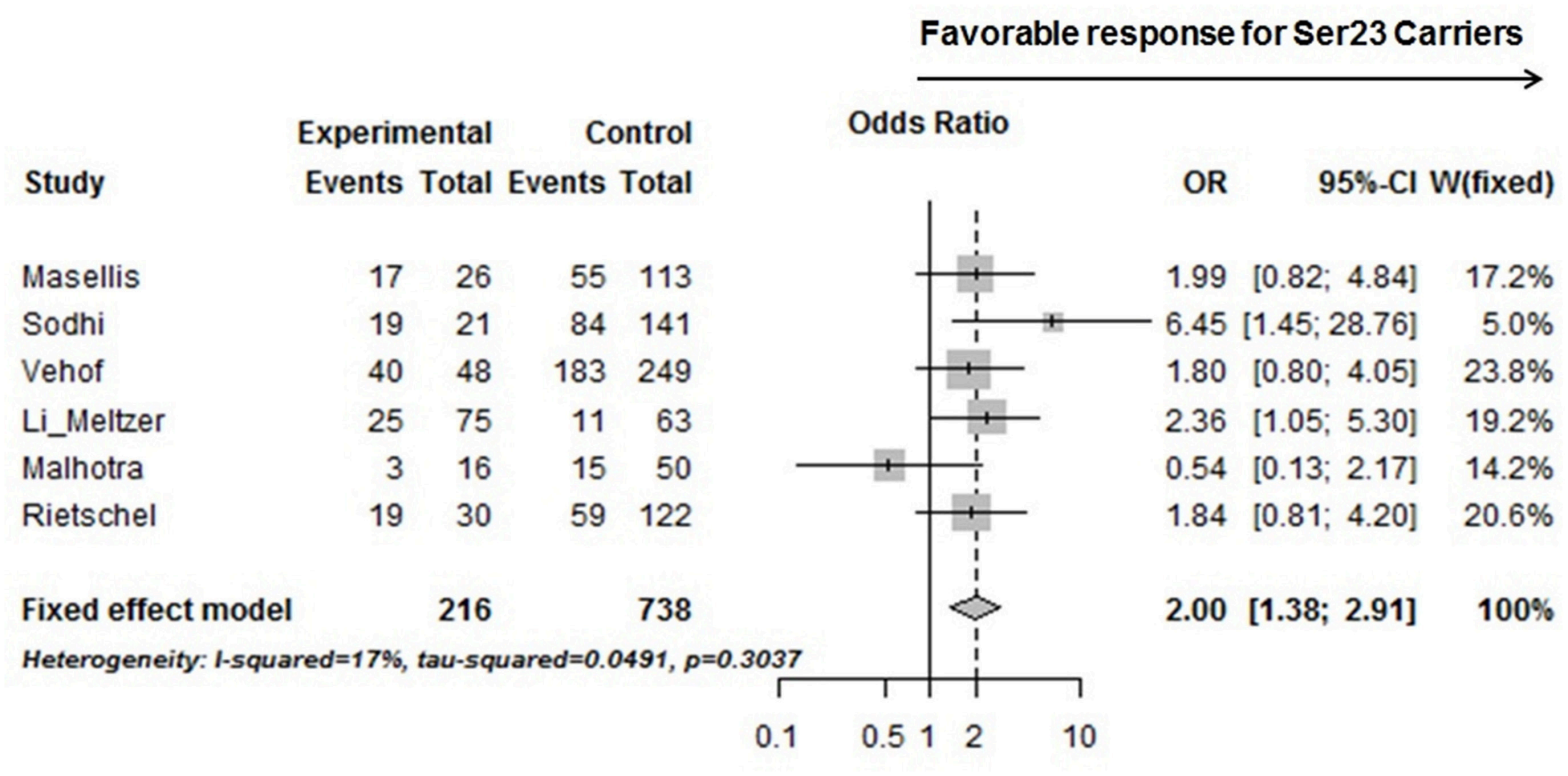
A forest plot showed a meta-analysis of the Ser23 carrier for rs6318 (Cys23Ser) associated with treatment response (binary data) in positive symptom across six studies on EUR samples. Experimental, Ser23 carrier; Control, Non-Ser23 carrier; Events, number of Responder; Total, number of subjects with that genotype. The squares represent odds ratio to have a favorable treatment response if it is over 1 and the horizontal lines show the 95% confidence intervals of the corresponding odds ratio. For Vehof's study, the raw data was imputed based on published improvement/no improvement ratio, genotyping data, and the corresponding odds ratio.

Six studies from Table 3, including ours, with accessible genotyping data for Cys23Ser and binary outcome for positive symptom improvement in EUR, were included in a meta-analysis. We reported the overall odds ratio is 2.00 (95%CI, 1.38–2.91, *p* = 0.0003) or 1.94 (95%CI, 1.27–2.99, *p* = 0.0024) under the fixed or random effect models, respectively (Figure 2). The heterogeneity between the studies was insignificant (Cochran's Q = 6.03, *p* = 0.30; *I*^2^ = 0.17 (95% CI, 0.00 to 0.62). QUANTO 1.2 was used to calculate the power of the test. The Ser23 carriers were found to have a frequency of 0.15 to 0.45 according to Table 3. As Ser23 carriers increased the odds of having treatment response by 2.0, population risk (*Kp)* = 0.30, dominant mode of inheritance, and 216 responders/738 non-responders were genotyped, the power (chance) to detect an association with significance *p* < 0.01 was over 90%.

**Table 3:** w studies to determine the general relationship between HTR2C Cys23Ser and drug response to APDs.

**Reference**	**Male/female**	**Antipsychotics**	**Ethnicity**	**Study duration**	**Genetic variants**	**Responder/no*n*-responder**	**trait**	**Statistical analysis**	**Summary of the result (*p* < 0.05, uncorrected for multiple testing)**
Sodhi et al.^*^ (33)	162 (unclear)	Clozapine only	Caucasian	3 months	rs6318	103/59	Global assessment scale. 20 point improvement as cutoff for response (binary trait) or raw changes (quantitiative trait)	Chi-square, Anova test. hemizygous males and homozygous females grouped together	Anova, *p* = 0.002; Dominant mode, *X*^2^ = 7.7, *p* = 0.005
Masellis et al.^*^ (37)	185 (132/53)	Clozapine only	Caucasian /African-American	6 months	rs6318	72/67 for Caucasian; 20/19 for Afrian Americans	A reduction of ≥20% in total BPRS score (binary trait)	Chi-square, separate Caucasian and AA, hemizygous males and homozygous females grouped together	*X*^2^ = 3.46, *p* = 0.18 for Caucasians; *X*^2^ = 0.31, *p* = 0.86 for African-American
Arranz et al. (34)	200 (unclear)	Clozapine only	Caucasian	3 months	−330(GT)/-244(CT); rs6318	133/67	Global assessment scale. 20 point improvement as cutoff for response (binary trait) or raw changes (quantitiative trait)	Dominant genetic model. Chi-square. hemizygous males and homozygous females grouped together	Genotypic association, *p* = 0.04 for 330(GT)/224(CT); *p* = 0.08 for rs6318
Reynolds et al. (18)	117 (58/59)	Chlorpromazine (56.4%); risperidone (36.8%); clozapine (3.4%); fluphenazine (2.6%); sulpiride (1%)	Chinese (Han)	2.5 months	rs3813929	86/90	% change of PANSS (positive, negative, general) (quantitative trait); A reduction of ≥50% in total PANSS score (binary trait)	Anova test to determine any association with genotype of the percentage change in PANSS score. Any sigificant result was then followed by analysis to determine which subscores of PANSS contribute to the overall difference. Stepwise regression analysis to determine the the influence of clinical and demographis factors on symptom measures.	Anova, *p* = 0.023 for negative subscore; *p* = n.s. for postive subscore. Chi-square, When separate male and female, this significant association only in male subjects (*p* = 0.007)
Ikeda (44)	120 (58/62)	Risperidone only	Japanese	2.5 months	rs3813929; rs518147	Not available	% change in PANSS (positive, negative, general) (quantitative trait)	Dominant genetic model. Anova test	*p* = 0.315 for rs3813929; *p* = 0.222 for rs518147
Need (45)	524 (CATIE Phase 1)	Olanzapine, perphenazine, quetiapine, risperidone and ziprasidone	Caucasian /African-American	3 months	30 tag SNPs in HTR2C	Not available	Delta change of PANSS (positive, negative, general) (quantitative trait)	All drug groups analyzed together using linear regression with additive genetic model and including eigenstrat axis, sex, self-described race, baseline, and phase I drug as covariates	Not significant; Data not available
Liu et al. (19)	130 (45/85)	Risperidone only	Chinese (Han)	2 months	rs3813929: rs518147; rs1023574; rs9698290; rs6318	Not available	% change of total BPRS score (quantitative trait)	Anova test for the genetic association with risperidone efficiency in the female and male group, respectively. Dominant genetic model to the regression analysis.	Anova, Male/Female, *p* = 0.533/0.006 for rs518147; *p* = 0.676/0.062 for rs3813929; *p* = NA for rs6318 due to low MAF
Vehof et al.^*^ (46)	329 (260/69); actual number is 293 (-/-) for rs3813929 and 297 for rs6318.	Clozapine (9.1%); Olanzapine (24.3%); Risperidone (22.8%); Quetiapine (5.5%); Haloperidol (7.3%); Multiple (11.2%); Aripiprazole (1.5%); others (6.7%)	Caucasian	variable	rs3813929; rs6318	247/82	Clinical Global Impression—Improvement scale positive symptoms (binary and quantitative trait)	Ordinal regression analysis (additive model) corrected for age and gender. hemizygous males and homozygous females grouped together.	β = 0.08, *p* = 0.79 for rs3813929; β = −0.47, *p* = 0.13 for rs6318. a negative β or odds ratio <1 means more improvement (lower CGI score) per extra minor allele. A trend is observed.
Malhotra et al.^*^ (35)	66 (49/17)	Clozapine only	Caucasian	2.5 months	rs6318	18/48	A reduction of ≥20% in total BPRS score (binary trait)	Fisher Exact test	*p* = 0.30
Rietschel et al.^*^ (36)	152 (76/76)	Clozapine only	Caucasian	variable (Avg = 2.0 months)	rs6318	110/42	Self defined four different groups from group 0 (worsening/no change), 1 (slight improvement), 2 (marked improvement), 3 (total reduction)	Chi-square separated by male and female.	*X*^2^ = 2.161, *p* = 0.142. A trend of Ser in responder (24%) than in non-responder (15%)
Li and Meltzer^*^	171 (115/56)	Clozapine (78%); Olanzapine (2.1%); Risperidone (3.8%); Melperone (7.0%); Others (9.0%)	Caucasian /African-American	6 weeks & 6 months	rs3813929; rs518147; rs6318	74/63 (in terms of positive symptom at 6 month)	A reduction of ≥20% in total BPRS score (binary trait); absolute change (Δ) in BPRS total score and subscales (quantitative trait)	Chi-square separated by male and female; Linear regression adjusting for covariates; ANCOVA adjusting for covariates. Individual SNP association and Haplotype association tests	In male patients, *X*^2^ = 7.54, *p* = 0.01 for rs6318 in association with positive symptoms response at 6mon; *X*^2^ = 7.80, *p* = 0.03 in association with negative symptoms response at 6mon.

Previous association studies of HTR2C polymorphisms with treatment response to antipsychotics, mainly clozapine, in Schizophrenia.

* *represents studies included in the meta-analysis*.

## Discussion

We tested the association between the HTR2C polymorphisms, Cys23Ser, −759C/T, and −697G/C, and treatment response in 171 schizophrenic patients after treatment with APDs, mainly clozapine, for 6 months. One of the strengths of this study was that the majority of the patients were unmedicated at the time of initial assessment. Both Ser23 and −759C–Ser23 haplotype were significantly associated with positive and negative symptom improvement in male patients and Cys23Ser, not the promoter polymorphisms, is the major genetic contributor of the HTR2C in modulating symptom improvement to clozapine. The previously published association studies (Table 3) no consistent results. Attempted replication studies (37, 46) for Sodhi et al.'(33) reported negative results, but the individual *p*-values as well as the *p*-value from a meta-analysis (47) were suggestive of a trend for association (Masellis, *p* = 0.18, Vehof, *p* = 0.13, and Gressier, *p* = 0.12). Our meta-analysis of six original studies (33, 35–37, 46) suggests that HTR2C Cys23Ser is associated with symptom improvement after treatment with clozapine. This is consistent with a previous meta-analysis that included several APDs (48).

Some atypical APDs, e.g., clozapine, olanzapine, risperidone, and sertindole, are potent inverse agonists of both HTR2C and HTR2A receptors (49, 50). On the other hand, some typical APDs, e.g., chlorpromazine, thioridazine, spiperone, and thiothixene, are HTR2C neutral antagonists, which would preclude their affecting the constitutive activity of HTR2Cs, although the combination of a neutral antagonist and inverse agonist, could lead to blockade of the neutral antagonist (51). The Ki (nM) for D2, HTR2A, and HTR2C are provided in Supplementary Table 5 for each APD (51). Studies based mainly on a single APD are more likely to generate positive results than those based on diverse drug treatments because APDs have variable effects on non-5-HT2C, receptors which can affect their actions as antipsychotics and cognitive enhancers (49, 50). However, the meta-analysis reported here suggests our findings may generalize to atypical APDs which are HTR2C antagonists or inverse agonists at clinically effective doses.

Many factors may contribute to the inconsistent results in pharmacogenetic studies of APDs response. These include the heterogeneity in patient populations, utilization of different rating scales, definition of response, frequency of genetic variants in distinct ethnic groups, APDs which differ with regard to HTR2C pharmacology and actions on other receptors which impact response, e.g., D2, 5-HT1A, and alpha 2 adrenoreceptors, duration of clinical assessment, proposed mode of inheritance, and statistical methods.

*In vitro* functional studies provide a partial explanation of why Cys23Ser has a main effect on response to APD treatment. Ser23 receptor displayed greater constitutive activity to mobilize calcium than Cys23 receptor (52). Ser23 receptor had greater cell surface expression and more rapid resensitization following exposure to SB206553, a mixed HTR2B antagonists and HTR2C inverse agonist (53). It may be concluded that prolonged exposure of both HTR2C isoreceptors to an inverse agonist increases receptor responsiveness to endogenous 5-HT or other HTR2C agonists, and cells or presumably individuals carrying Ser23 have prompter response to the stimuli than Cys23 carriers. Dopaminergic circuitry is more sensitive to pain stress in Ser23 carriers (54). Greater dopamine release in the nucleus accumbens, caudate nucleus, and putamen was observed in the Ser23 carriers during pain, suggesting mesoaccumbal stress sensitivity may mediate the effects of HTR2C variation on the risk of neuropsychiatric disorders. Significant differences in regional cerebral blood flow between Ser23 and Cys23 male carriers after treatment with serotonin agonist meta-Chlorophenylpiperazine suggests that this polymorphism does have distinct functional consequences (55).

In conclusion, these results provide additional evidence that HTR2C polymorphisms, particularly Cys23Ser, are associated with response to APD treatment, mainly clozapine with HTR2C antagonism or partial agonism, in male schizophrenic patients.

## Ethics Statement

This study was carried out in accordance with the recommendations of Internal Review Board of Vanderbilt University and Case Western Reserve University with written informed consent from all subjects. All subjects gave written informed consent in accordance with the Declaration of Helsinki. The protocol was approved by the Internal Review Board of Vanderbilt University and Case Western Reserve University.

## Author Contributions

JL mostly contributed to data analysis, interpretation of results, and manuscript writing. HH contributed to initial data analysis and manuscript writing. HM has designed the study and written the manuscript in close collaboration with JL. All authors have approved the final manuscript.

### Conflict of Interest Statement

HM is a stockholder in SureGene and ACADIA and receives additional grant support from Sunovion and Sumitomo Dainippon Pharma for other studies. HM also receives grant support from Alkermes, Auspex, Boehringer Mannheim, Eli Lilly, Janssen, Lundbeck, Mag T, Otsuka, and Reviva. The remaining authors declare that the research was conducted in the absence of any commercial or financial relationships that could be construed as a potential conflict of interest.
