# Supplementary material for: Association of Serotonin2c Receptor Polymorphisms With Antipsychotic Drug Response in Schizophrenia
Source: Front Psychiatry. 2019 Feb 15;10:58. doi: 10.3389/fpsyt.2019.00058 (PMC6384235; doi:10.3389/fpsyt.2019.00058)
Supplement: Supplementary file 1 [file Data_Sheet_1.pdf]

Supplementary Material

Supplemental Information for the Method

1. Clinical evaluation of treatment response

The majority of these patients were initially hospitalized for an acute exacerbation of symptoms or because of failure to respond adequately to conventional or atypical antipsychotic drugs between 1995 and 2010, leading to 118 patients (69%) with initial diagnosis of treatment-resistant schizophrenia (TRS) as defined by Kane, et al(Kane et al., 1988). Demographic information is provided in **Table 1** separated by gender. Over 75% of patients were unmedicated or had a drug free period of 3-10 days prior to baseline assessment. Patients were interviewed by trained raters using the Schedule for Affective Disorders and Schizophrenia(Endicott and Spitzer, 1978) to establish diagnosis. This was integrated with all available data to make the final diagnosis by consensus according to the Diagnostic and Statistical Manual of Mental Disorders, third edition (DSM-III) criteria at discharge. A review of these diagnoses indicates all patients meet DSM-IV criteria for schizophrenia or schizoaffective disorder. For the purpose of this report, data from patients with either diagnosis are combined. Brief Psychiatric Rating Scale (BPRS; items rated 0-6)(Overall and Gorham, 1962) was used to assess the severity of psychopathology. BPRS positive symptom subscale includes suspiciousness, hallucinatory behavior, unusual thought content, and conceptual disorganization. In a factor analysis of BPRS ratings in 572 schizophrenia patient, including the subjects in this study, conceptual disorganization did not cluster with suspiciousness, hallucinatory behavior, and unusual thought content (Jayathilake et al unpublished data). Similar findings have been reported by others (Lindenmayer et al., 1994), suggesting conceptual disorganization is weakly connected to the positive symptom domain. We, therefore, excluded conceptual disorganization in the analyses of positive symptoms which follow. The remaining three items, suspiciousness, hallucinatory behavior, and unusual thought content are referred to as the positive 3-item. BPRS negative subscale is comprised of three items: emotional withdrawal, motor retardation and blunted affect. The anxiety-depression subscale consists of three items as well: anxiety, guilty feelings, and depressive mood. Categorical treatment response was

evaluated at 6 week and 6 months, using the criteria based upon Kane et al(Kane et al., 1988). A reduction of > 20% in BPRS total or subscale scores was considered as responder. In cases where patients were very close to the operational criteria for response (> 15% or < 25%), a reduction of at least one category on the Clinical Global Impressions (CGI) scale was considered in order to enhance the definition of response.

After a description of the study, written informed consent was obtained from every subject. All patients provided written informed consent to remain drug free during the assessment. The drug free period was terminated if patient well-being required it. Some were not receiving psychotropic drugs prior to admission because of non-compliance. The study protocol was approved by institutional ethics committees and was performed in accordance with the ethical standards laid down in the 1964 Declaration of Helsinki.

2. ***Mapping cis eQTL and methylation QTL***

BrainCloud allows the query of genome-wide gene expression data and their genetic control in human postmortem dorsolateral prefrontal cortex of normal subjects across the lifespan(Colantuoni et al., 2011). We included all the SNP loci available within 200kb interval (100kb up- and downstream) of HTR2C transcript. The *p* value corresponds to the regression coefficient based on the residual expression level from the General Linear Model and the genotype obtained from 110 postnatal Caucasian subjects. We also inquire a genome-wide DNA methylation database, BrainCloud Methyl, derived from the same BrainCloud subjects(Numata et al., 2012), to see if those identified *cis* eQTLs have any impact on DNA methylation and considered as methylation QTLs.

3. ***Statistical analysis***

We analyzed the males and females separately because of functional uncertainty in heterozygous females due to either X chromosome inactivation (XCI) or other confounding factors related to gender difference, for example, a much stronger effect of the genetic association with clozapine-induced weight gain was observed in female subjects(Covell et al., 2004).

The relationship between genotypes and demographic variables was analyzed using chi-square ($\chi2$) or ANOVA. *P*-values reported are two-tailed whenever applicable. Haplotype association analysis was performed for all possible combinations of SNPs (including consecutive and non-consecutive SNPs). For rs6318, genotypes were collapsed into a dominant model, separated by Ser carrier or non-carrier. Genotypes/haplotypes effects on the binary outcome were analyzed at 6 weeks, and 6 months by comparing the frequencies of alleles or genotypes between responder and non-responder groups using the following methods: Pearson's Chi-square and ANCOVA which was also performed to test the association between HTR2C polymorphisms and percentage change (%) or absolute change (Δ) in BPRS total score and subcategories, after controlling for race, drug, age of onset, and the corresponding baseline psychopathology. Statistical significance was defined as $p < 0.05$. As all results are considered exploratory, there was no adjustment for multiple comparisons.

Linkage disequilibrium (LD) was visualized by Haploview. SNP Annotation and Proxy Search (SNAP) was used to search proxy SNPs for target SNPs. Genotyping data from 1000Genome Database was extracted to confirm those tag SNPs were indeed representative.

4. **Meta-analysis**

In order to review and elucidate the general relationship between HTR2C polymorphisms and drug response to APDs, a Pubmed search was conducted using Medline databases from 1966 to February, 2016. The following combination of search terms, "antipsychotic agents"[MeSH Terms] AND 5-HT2C[All Fields] OR HTR2C[All Fields] AND "humans"[MeSH Terms], help to identified 217 abstracts. There are 11 English-language citations which were extracted because of the topic on drug response in schizophrenia with reported genotype data on HTR2C polymorphisms. The references

listed from obtained articles were also searched to identify further relevant citations. There was a lack of study of rs6318 in East Asian population and rs3813929 in AFR population due to very low MAF (<5%, **Supplementary table 2**). As showed in **Table 2**, the following information was collected from these papers: names of first authors with year of publication, number of subject (separated by gender), percentage of patients on clozapine or other APDs, ethnicity (EUR, AFR, and East-Asian), study duration, observed genetic variants in HTR2C, Responder/Non-responder Ratio, Trait (phenotype observed as binary and/or quantitative), Statistical analysis, and summary of the results. Those full-text articles were then scrutinized by two authors, J Li and HY Meltzer, to determine eligibility for inclusion in the meta-analysis. Finally, six studies, including ours, with accessible genotyping data for rs6318 and binary outcome for symptom improvement, were included. We did not stratify the patients for each study based on the gender or ethnicity. Only two studies (ours and Masellis') recruited a small portion of AFR subjects. This is an extension of a previously reported meta-analysis on the same topic(Kirchheiner et al., 2004). Although over 50% of the subjects received clozapine or olanzapine treatment in each study except Vehof's, the definition of drug responder varied. We did not include a candidate-gene study of SNP array data generated from the CATIE (Clinical Antipsychotic Trials of Intervention Effectiveness) sample(Need et al., 2009) because clozapine was not one of the observed APDs and the trait for treatment response is quantitative, but not binary (**Table 2**). There is no significant deviation from HWE (p>0.001) for rs6318 in any of the studies. In order to reduce the heterogeneity among these studies and avoid publication bias, we included all patients (male and female); only improvement in positive symptoms was considered in our study in order to match another study(Vehof et al., 2012). Meta-analysis was performed by R 'meta' package. Heterogeneity among the studies was assessed by means of the I^2 inconsistency test and Cochran's Q statistics under a null hypothesis test in which $p < 0.05$.

Reference:

Colantuoni, C., Lipska, B.K., Ye, T., Hyde, T.M., Tao, R., Leek, J.T., Colantuoni, E.A., Elkahloun, A.G., Herman, M.M., Weinberger, D.R., and Kleinman, J.E. (2011). Temporal dynamics and genetic control of transcription in the human prefrontal cortex. *Nature* 478**,** 519-523.

Covell, N.H., Weissman, E.M., and Essock, S.M. (2004). Weight gain with clozapine compared to first generation antipsychotic medications. *Schizophr Bull* 30**,** 229-240.

Endicott, J., and Spitzer, R.L. (1978). A diagnostic interview: the schedule for affective disorders and schizophrenia. *Arch Gen Psychiatry* 35**,** 837-844.

Kane, J., Honigfeld, G., Singer, J., and Meltzer, H. (1988). Clozapine for the treatment-resistant schizophrenic. A double-blind comparison with chlorpromazine. *Arch Gen Psychiatry* 45**,** 789-796.

Kirchheiner, J., Nickchen, K., Bauer, M., Wong, M.L., Licinio, J., Roots, I., and Brockmoller, J. (2004). Pharmacogenetics of antidepressants and antipsychotics: the contribution of allelic variations to the phenotype of drug response. *Mol Psychiatry* 9**,** 442-473.

Lindenmayer, J.P., Bernstein-Hyman, R., and Grochowski, S. (1994). A new five factor model of schizophrenia. *Psychiatr Q* 65**,** 299-322.

Need, A.C., Keefe, R.S., Ge, D., Grossman, I., Dickson, S., Mcevoy, J.P., and Goldstein, D.B. (2009). Pharmacogenetics of antipsychotic response in the CATIE trial: a candidate gene analysis. *Eur J Hum Genet* 17**,** 946-957.

Numata, S., Ye, T., Hyde, T.M., Guitart-Navarro, X., Tao, R., Wininger, M., Colantuoni, C., Weinberger, D.R., Kleinman, J.E., and Lipska, B.K. (2012). DNA methylation signatures in development and aging of the human prefrontal cortex. *Am J Hum Genet* 90**,** 260-272.

Overall, J.E., and Gorham, D.R. (1962). The Brief Psychiatric Rating-Scale. *Psychological Reports* 10**,** 799-812.

Vehof, J., Burger, H., Wilffert, B., Al Hadithy, A., Alizadeh, B.Z., and Snieder, H. (2012). Clinical response to antipsychotic drug treatment: association study of polymorphisms in six candidate genes. *Eur Neuropsychopharmacol* 22**,** 625-631.

Supplementary Table 1 Summary of genotypic distribution of three mostly investigated HTR2C SNPs in EUR from 1000Genome, Braincloud, and our sample. Three datasets were genotyped by Illumina next-generation sequencing, Illumina Beadarray, and ABI Taqman assay, respectively. Data was presented as genotype count (% in Female or Male subjects).

Supplementary Table 2. A list of potential cis-eQTLs and methylation-QTLs identified by Braincloud and Brainmethyl database.

MAF = minor allele frequency. M = male; F = female. AFR = subjects with African ancestry; EUR = subjects with European ancestry. NA represents data not available or not reported due to low MAF resulting in unreliable data. $r^2>0.85$ in LD. SNPs are located in promoter region and putative CpG Island. Cys23Ser(rs6318) and its tag SNP, rs5987834, exclusively available in Braincloud were negative controls. $p<0.01$ is cut-off value for cis-eQTLs or methylation-QTL. P value between 0.01 and 0.05 suggests a possible impact of that SNP on gene expression or methylation.

Supplementary Table 3. Haplotype association analysis of HTR2C SNPs with treatment response by Chi-square test in female patients and all patients.

Supplementary Table 4. Ser23 carriers have a better symptom improvement in clozapine treated subjects. Male and female subjects were analyzed separately or together. All data was presented as Mean ± SD for Δ change (absolute change), which is calculated by (6wk or 6mon -Baseline); [&] represents F statistic and p value calculated from ANCOVA on Δ change at 6wk or 6mon after controlling for race, and the corresponding baseline psychopathology; * represents chi-square test on binary trait of symptom improvement defined as responder or non-responder. No significant association between -759C/T or -697 G/C and symptom improvement was observed (data not show).

Supplementary Table 5. Ki (nM), the inhibitor constant, for D2, HTR2A and HTR2C were collected from PDSP Ki database for each antipsychotic drugs (https://pdsp.unc.edu/databases/kidb.php)

Supplemental Table 1

-759C/T(rs3813929)	Female			Male		
**Data source**	**TT**	**TC**	**CC**	**T**	**C**	**tag SNP**
**1000 genome**	2 (1%)	56 (27.9%)	143 (71.1%)	32 (18%)	146 (82%)	rs3813929
**Braincloud**	1 (3.1%)	5 (15.6%)	26 (81.3%)	17 (21.8%)	61 (78.2%)	rs12846241
**Our sample**	3 (10.3%)	0(0%)	26 (89.7%)	12 (15.2%)	67 (84.8%)	rs3813929
**-697G/C(rs518147)**	**Female**			**Male**		
**Data source**	**CC**	**CG**	**GG**	**C**	**G**	**tag SNP**
**1000 genome**	16 (8%)	92(45.8%)	93 (46.3%)	67 (37.6%)	111 (62.4%)	rs498207
**1000 genome**	17 (8.4%)	91(45.3%)	93 (46.3%)	66 (37.1%)	112 (62.9%)	rs518147
**Braincloud**	3 (9.4%)	12 (37.5%)	17 (53.1%)	39 (50%)	39 (50%)	rs498207
**Our sample**	3 (12%)	8 (32%)	14 (56%)	23 (33.8%)	45 (66.2%)	rs498207
**Our sample**	6 (16.2%)	16 (43.2%)	15 (40.6%)	24 (30.4%)	55 (69.6%)	rs518147
**Cys23Ser(rs6318)**	**Female**			**Male**		
**Data source**	**CC**	**CG**	**GG**	**C**	**G**	**tag SNP**
**1000 genome**	4 (2%)	51 (25.4%)	146 (72.6%)	32 (18%)	146 (82%)	rs6318
**Braincloud**	2 (6.7%)	4 (13.3%)	24 (80%)	19 (24.4%)	59 (75.6%)	rs5987834
**Our sample**	2 (4.8%)	12 (28.5%)	28 (66.7%)	14 (16.7%)	70 (83.3%)	rs6318

Supplemental Table 2

SNP ID	Minor Allele	Observed Genotype	Location	SNP in promoter	MAF (M/F)	Tag SNP	p value for Cis-eQTL (AA/EUR)	p value for Methylation-QTL (AA/EUR)
**rs489736**	A	A/G	Intergenic-5’	No	0.382/0.316		0.058/0.045	0.002/0.002
**rs11798015**	G	G/A	Intergenic-5’	No	0.152/0.154	rs3813929	NA/0.004	NA/0.000
**rs547617**	G	G/A	Intergenic-5’	No	0.371/0.313	rs518147	0.162/0.043	0.003/0.021
**rs3795182**	G	G/A	near-gene-5’	Yes	0.185/0.149		NA	NA
**rs521018**	G	G/T	near-gene-5’	Yes	0.376/0.309		NA	NA
**rs498207**	G	G/A	near-gene-5’	Yes	0.376/0.309	rs518147	0.267/0.043	0.014/0.096
**rs3813928**	A	A/G	near-gene-5’	Yes	0.180/0.149	rs3813929	NA	NA
**rs3813929**	T	T/C	near-gene-5’	Yes	0.180/0.149	-759 (C/T)	NA	NA
**rs518147**	C	C/G	untranslated-5’	Yes	0.371/0.311	-697 (G/C)	NA	NA
**rs12846241**	G	G/T	intron	No	0.180/0.152	rs3813929	0.116/0.006	0.205/0.009
**rs12690355**	G	G/A	intron	No	0.191/0.154	rs3813929	NA/0.016	NA/0.028
**rs6318**	C	C/G	exon	No	0.180/0.147	cys23ser	NA	NA
**rs4911871**	G	G/A	intron	No	0.202/0.179		NA/0.009	NA/0.002
**rs6644093**	T	T/G	intron	No	0.146/0.132		NA/0.004	NA/0.007
**rs5987834**	T	T/C	Intergenic-3’	No	0.185/0.142	rs6318	0.914/0.601	0.561/0.944

Supplemental Table 3

Female Only

SNP ID	Haplotype	Haplotype frequency	BPRS Positive 4 Items		BPRS Positive 3 Items		BPRS Negative 3 Items	
			Frequency in Responder/non-responder	X^2/P	Frequency in Responder/non-responder	X^2/P	Frequency in Responder/non-responder	X^2/P
**rs3813929 (-759)**	C	0.936	0.95/0.846	2.056/0.152	0.95/0.833	2.403/0.121	0.833/0.941	1.764/0.184
**rs518147 (-697)**	C	0.364	0.333/0.438	0.889/0.346	0.326/0.438	1.003/0.317	0.385/0.370	0.0161/0.899
**rs6318 (Cys23Ser)**	Ser carrier	0.397	0.296/0.438	1.764/0.184	0.32/0.375	0.263/0.608	0.4667/0.25	**3.9/0.048**
**rs3813929-rs518147**	C-C	0.25	0.211/0.231	0.037/0.847	0.211/0.208	0.000/0.984	0.136/0.235	0.828/0.363
**rs518147-rs6318**	C-Ser	0.213	0.188/0.219	0.117/0.732	0.196/0.188	0.008/1	0.192/0.174	0.038/0.846
**rs3813929-rs6318**	C-Ser	0.413	0.3/0.462	1.777/0.183	0.3/0.417	0.905/0.341	0.417/0.294	0.935/0.333
**rs3813929-rs518147-rs6318**	C-C-Ser	0.205	0.18/0.219	0.187/0.666	0.188/0.188	0/1	0.179/0.174	0.003/0.959

Male + Female

SNP ID	Haplotype	Haplotype frequency	BPRS Positive 4 Items		BPRS Positive 3 Items		BPRS Negative 3 Items	
			Frequency in Responder/non-responder	X^2/P	Frequency in Responder/non-responder	X^2/P	Frequency in Responder/non-responder	X^2/P
**rs3813929 (-759)**	C	0.894	0.878/0.868	0.0416/0.839	0.8970.845	0.937/0.333	0.836/0.865	0.245/0.621
**rs518147 (-697)**	C	0.349	0.389/0.326	0.774/0.379	0.387/0.329	0.623/0.43	0.397/0.327	0.834/0.361
**rs6318 (Cys23Ser)**	Ser carrier	0.305	0.293/0.261	0.231/0.631	0.33/0.215	2.89/0.089	0.377/0.183	**8.122/0.004**
**rs3813929-rs518147**	C-C	0.22	0.238/0.169	1.195/0.274	0.259/0.141	3.302/0.069	0.203/0.157	0.520/0.471
**rs518147-rs6318**	C-Ser	0.194	0.214/0.159	0.863/0.353	0.239/0.127	3.539/0.060	0.222/0.13	2.378/0.123
**rs3813929-rs6318**	C-Ser	0.294	0.284/0.256	0.160/0.689	0.314/0.211	2.093/0.148	0.35/0.171	**6.248/0.012**
**rs3813929-rs518147-rs6318**	C-C-Ser	0.187	0.206/0.157	0.737/0.391	0.231/0.126	3.181/0.075	0.203/0.128	1.629/0.202

Supplementary Table 4.

Group	Phenotype	Duration of treatment	Ser Carrier Frequency	Number of Subject	Cys23Ser(rs6318)		ANCOVA&	Chi-square*
					Ser Carrier	Ser non-Carrier	*F/P*	*X^2/P*
Male	Positive 3 items	6 month	0.241	87	-4.14±3.90	-1.45±4.32	**4.017/0.048**	**4.845/0.028**
		6 week	0.234	94	-2.64±4.12	-1.58±4.04	0.177/0.675	0.824/0.364
	Negative 3 items	6 month	0.233	90	-2.10±2.91	-0.43±3.22	**5.088/0.027**	**4.375/0.036**
		6 week	0.245	98	-0.54±2.30	-0.03±3.24	0.349/0.556	0.129/0.719
Female	Positive 3 items	6 month	0.368	38	-2.93±3.54	-3.67±3.96	0.793/0.379	0.015/0.901
		6 week	0.395	43	-2.94±4.44	-2.35±3.79	0.139/0.711	0.269/0.604
	Negative 3 items	6 month	0.385	39	-2.33±3.48	-0.83±2.94	**4.793/0.035**	1.262/0.261
		6 week	0.409	44	-1.78±3.06	-0.77±3.01	2.906/0.096	**4.174/0.041**
Both	Positive 3 items	6 month	0.278	126	-3.66±3.76	-2.04±4.32	1.171/0.281	**4.474/0.034**
		6 week	0.285	137	-2.77±4.21	-1.79±3.97	0.174/0.678	0.309/0.578
	Negative 3 items	6 month	0.279	129	-2.19±3.12	-0.54±2.99	**8.208/0.005**	**5.784/0.016**
		6 week	0.296	142	-1.07±2.69	-0.22±3.18	2.127/0.147	3.141/0.076

Supplemental Table 5

APDs	Ki(nM)		
	D2	HTR2A	HTR2C
**Clozapine**	130	6.025	12.1
**Olanzapine**	20.5	2.83	12.5
**Risperidone**	2.45	0.36	32
**Melperone**	194	230	2100
Amisulpride	2.15	8304	10000
Aripiprazole	2.125	21.85	22.4
Iloperidone	3.3	0.16	42.8
Lurasidone	1.68	2	415
Quetiapine	212.5	169.8	1400
Asenapine	1.3	0.07	0.03
Sertindole	2.7	0.3	3.45
Zotepine	16.5	2.65	3.2
Chlorpromazine	2.65	2.78	15.55
Spiperone	0.1	1.25	746
Thioridazine	10	12.55	53
thiothixene	0.415	50	1355.5
